# Comparative Efficacy and Safety of Tralokinumab and Dupilumab in Moderate-to-Severe Atopic Dermatitis: A Narrative Review

**DOI:** 10.3390/jcm14144960

**Published:** 2025-07-13

**Authors:** Yoon-Seob Kim

**Affiliations:** Department of Dermatology, Bucheon St. Mary’s Hospital, College of Medicine, The Catholic University of Korea, Seoul 06591, Republic of Korea; kysbbubbu@catholic.ac.kr; Tel.: +82-32-340-2115; Fax: +82-32-340-2118

**Keywords:** atopic dermatitis, tralokinumab, dupilumab, biologic therapy, efficacy and safety

## Abstract

Atopic dermatitis (AD) is a chronic inflammatory skin disorder that significantly affects patients’ quality of life. Dupilumab, a monoclonal antibody targeting interleukin (IL)-4 receptor alpha (IL-4Rα), has been the standard biologic therapy for moderate-to-severe AD. This review compares dupilumab with tralokinumab—a promising alternative that selectively neutralizes IL-13—by examining their distinct mechanisms, clinical efficacy, safety profiles, and practical considerations. While both biologics are highly effective, pivotal monotherapy trials indicate numerically higher efficacy rates for dupilumab. Regarding safety, while long-term data show comparable rates of serious adverse events, dupilumab is associated with a higher incidence of both conjunctivitis and injection-site reactions. Key practical differences include dupilumab’s broader indications and approval for infants (≥6 months), versus tralokinumab’s flexible maintenance dosing and notable efficacy in head and neck AD. By highlighting these key distinctions, this review aims to support personalized treatment selection in AD. However, no direct head-to-head clinical trials have yet compared dupilumab and tralokinumab, and the available evidence is based on indirect comparisons from separate pivotal studies.

## 1. Introduction

Atopic dermatitis (AD) is a chronic, relapsing inflammatory skin disease characterized by eczematous lesions, intense pruritus, and impaired skin barrier function with a lifetime prevalence of 15–20% in developed countries [1]. The pathogenesis of AD is complex and multifactorial, involving genetic predisposition, barrier abnormalities, immune imbalance, and microbial dysbiosis, ultimately contributing to chronic inflammation with pruritus and atopic comorbidities [1,2,3,4,5,6,7]. Recent evidence also highlights the role of the skin as a neuro-immune-endocrine organ that integrates environmental influences, a perspective highly relevant to understanding AD pathophysiology [8]. In moderate-to-severe cases, AD imposes a significant psychosocial and economic burden, severely impacting patients’ quality of life. Traditional systemic treatments, such as corticosteroids and immunosuppressants, provide limited long-term efficacy and carry potential safety concerns, thus prompting the development of more targeted biologic therapies [9,10,11,12].

The therapeutic landscape of AD has rapidly evolved with the introduction of several biologic agents and small-molecule Janus kinase (JAK) inhibitors. Dupilumab, tralokinumab, and lebrikizumab are now considered among the most established biologic agents targeting type 2 cytokines [9,10,11]. JAK inhibitors, including baricitinib, abrocitinib, and upadacitinib, provide an oral alternative, with differences in onset of action, efficacy, and safety profiles [9,10,11]. Given the expanding therapeutic options, dermatologists now face increasing complexity in treatment selection, as even biologics targeting the same pathway differ markedly in their mechanisms, efficacy, safety, and usability.

Among these advanced therapies, dupilumab and tralokinumab have become the most widely used biologics for moderate-to-severe atopic dermatitis, supported by robust long-term safety data and growing real-world experience [13,14]. Although both agents target type 2 inflammation and have demonstrated efficacy in large-scale trials, they differ in mechanism of action, safety profile, dosing frequency, and administration method, all of which have important implications for clinical decision-making [15,16].

This review focuses on dupilumab and tralokinumab, as these biologics are currently the most widely approved and clinically utilized biologics for moderate-to-severe AD. Other novel targeted biologics, such as lebrikizumab (anti–IL-13 antibody), nemolizumab (anti–IL-31 receptor antibody), and rocatinlimab (anti–OX40 antibody), are emerging or have recently been approved in select regions, but robust real-world and comparative data remain limited. Furthermore, a direct, pairwise comparison provides a clearer and more clinically actionable understanding of the key differences in efficacy, safety, and practical aspects between dupilumab and tralokinumab. This approach enables clinicians to better appreciate the advantages and limitations of each drug and to apply this knowledge effectively in routine care.

## 2. Pathophysiology of Atopic Dermatitis: An Overview

The pathogenesis of AD is a multifaceted process sustained by a cycle involving (1) genetic predisposition, (2) barrier impairment, (3) immune dysregulation, (4) microbiome imbalance, and (5) neuro-immune crosstalk [1,2,3,4,5,6,7]. These interconnected domains interact to propagate dysfunction across systems, ultimately driving the chronic, relapsing nature of AD. Furthermore, AD is not merely a localized skin disorder but represents a systemic disease process that frequently coexists with or precedes other atopic comorbidities (asthma, allergic rhinitis, food allergy, and allergic conjunctivitis), a phenomenon often described as the atopic march [1,2,3,4,5,6,7]. AD typically begins in early childhood, with up to 85% of cases occurring before age 5, and about one-third to one-half of patients continue to be affected into adulthood, reflecting its chronic, relapsing nature [17]. Typical clinical features of AD include pruritus, eczematous lesions, flexural involvement, and generalized xerosis, which together form the cornerstone of commonly used diagnostic criteria [9,18].

Genetic predisposition represents a foundational aspect of AD pathogenesis, shaping epidermal barrier properties, immune response profiles, and apoptotic regulation. While loss-of-function mutations in the filaggrin (FLG) gene are the most extensively characterized and directly linked to increased transepidermal water loss and enhanced penetration of allergens and microbes [19], genome-wide association studies have underscored the broader complexity of genetic susceptibility [20,21]. A landmark multi-ancestry study involving over 21,000 cases and 95,000 controls not only reaffirmed the significance of the *FLG* locus—encompassing multiple genes within the epidermal differentiation complex that encode structural and antimicrobial proteins essential for both barrier integrity and innate immune defense—but also identified additional susceptibility loci [21]. Additional susceptibility loci identified included *OVOL1* (epidermal differentiation) and *KIF3A* (epithelial barrier function), *IL13* (type 2 cytokine signaling), and *TNFRSF6B* (apoptosis regulation), highlighting the complex polygenic architecture shaping the barrier, immune, and apoptotic pathways implicated in AD susceptibility [21]. Collectively, these genetic determinants establish a multifaceted susceptibility framework that simultaneously influences barrier integrity, immune polarization, and cell survival pathways, thereby priming individuals for the development of AD upon environmental challenge.

Epidermal barrier dysfunction often represents the earliest clinical expression of genetic and environmental susceptibility in AD [22]. Structural compromise of the skin, whether genetically determined or exacerbated by external factors such as allergens (e.g., house dust mite), detergents and other irritants, pollutants, climate fluctuations, or psychological stress via local HPA axis activation, increases transepidermal water loss and facilitates the penetration of allergens, microbes, and other irritants [23]. Barrier disruption triggers the release of epithelial-derived alarmins, including TSLP, interleukin (IL)-33, and IL-25, which activate dendritic cells and initiate type 2 immune polarization characterized by Th2 and ILC2 expansion [24,25,26]. Co-stimulatory interactions such as OX40-OX40L engagement between dendritic cells and T cells further amplify this polarization and sustain chronic inflammation, contributing to the persistent nature of AD [24,25,26]. This close interaction between barrier disruption and immune activation underscores the bidirectional nature of AD pathogenesis.

Immune dysregulation, characterized by dominant type 2 cytokine signaling (IL-4, IL-5, and IL-13), not only perpetuates inflammation but also further impairs the skin barrier by downregulating structural proteins—including filaggrin, loricrin, and involucrin—and suppressing antimicrobial peptide production [24,25,26]. Although acute lesions are primarily governed by this type 2 axis, chronic AD often shows increased Th1, Th17, and Th22 activity, contributing to persistent inflammation and dermal remodeling [27,28,29]. Notably, studies have suggested that Asian patients with AD may exhibit relatively stronger Th17 and Th22 polarization, although the clinical significance remains to be fully elucidated [30,31]. Among the various immune pathways implicated in AD, the IL-4 and IL-13 signaling axis is recognized as the central driver in AD pathogenesis and will be discussed in detail in this review.

Microbial dysbiosis, particularly involving the skin and gut microbiome, represents a critical component of this multifactorial process, influencing both local and systemic manifestations of AD [32,33,34,35,36]. On the skin, lesions exhibit reduced microbial diversity and pronounced colonization by *Staphylococcus aureus*, identified in up to 90% of patients compared to less than 10% of healthy controls [37,38]. *Staphylococcus aureus* exacerbates disease by releasing superantigens and proteases that intensify type 2 inflammation and further impair barrier integrity [37,38]. Beyond the skin, the gut–skin axis underscores how intestinal dysbiosis—characterized by decreased populations of short-chain fatty acid-producing genera such as *Bifidobacterium* and *Faecalibacterium*—compromises regulatory T cell function and weakens systemic immune tolerance, fueling the broader atopic march [32,33,34,35,36]. Notably, gut microbiota alterations in AD have been more consistently reported compared to those of the skin microbiome, which are often confounded by sampling variability and site-specific factors [39,40]. Gut microbiota alterations in AD extend beyond mere increases in intestinal permeability, indicating a fundamental breakdown of gut-derived immunoregulatory networks [32,33,34,35,36].

Neuro-immune crosstalk also contributes importantly to AD pathogenesis, particularly in mediating chronic pruritus [41,42]. The skin serves as a dynamic interface integrating immune signals, peripheral nerve activity, and local stress pathways, all of which become dysregulated in AD [8]. Type 2 cytokines such as IL-4, IL-13, and IL-31 sensitize cutaneous sensory neurons, lowering their activation thresholds and fueling pruritus, which in turn provokes scratching that further disrupts the barrier, releases additional alarmins, and amplifies local immune responses [43,44,45]. Chronic inflammation and pruritus also promote hyperinnervation and morphological changes in epidermal nerve fibers, reflecting peripheral neuroplasticity [46]. Moreover, neuroimaging studies have demonstrated altered activation of the insular and anterior cingulate cortex in AD patients, suggesting that central sensitization contributes to itch persistence beyond cutaneous inputs [47]. Although the molecular underpinnings of these neuro-immune-endocrine alterations remain largely uncharted, they collectively underscore the complex multidirectional network sustaining AD.

**Figure 1:**
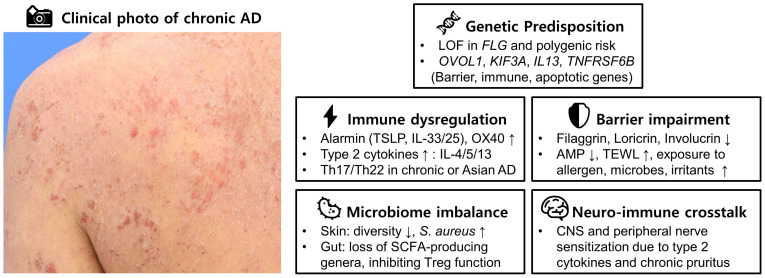
Clinical photograph of chronic atopic dermatitis with summary schematic of the five major pathogenic domains. Abbreviations: AD, atopic dermatitis; AMP, antimicrobial peptides; CNS, central nervous system; FLG, filaggrin; IL, interleukin; LOF, loss-of-function; OX40, tumor necrosis factor receptor superfamily member 4; SCFA, short-chain fatty acid; TEWL, transepidermal water loss; Th, T helper; Treg, regulatory T cell; TSLP, thymic stromal lymphopoietin.

The major pathogenic domains of atopic dermatitis—genetic predisposition, barrier impairment, immune dysregulation, microbiome imbalance, and neuro-immune crosstalk—are summarized together with a representative clinical photograph in Figure 1.

## 3. Mechanism of Action of Dupilumab and Tralokinumab

AD is a multifactorial disease involving genetic predisposition, skin barrier dysfunction, immune dysregulation, and microbial imbalance [1]. Filaggrin deficiency, altered lipid composition, and tight junction abnormalities compromise the epidermal barrier, facilitating allergen penetration and colonization by *Staphylococcus aureus* [1]. These events activate innate and adaptive immune responses, leading to chronic type 2 inflammation driven primarily by IL-4 and IL-13 [15]. These cytokines not only promote Th2 differentiation, IgE class switching, and eosinophil recruitment but also impair keratinocyte differentiation, suppress antimicrobial peptides, and perpetuate barrier dysfunction [24]. The interplay between immune activation and barrier impairment creates a self-amplifying inflammatory loop, positioning IL-4 and IL-13 as central, yet functionally distinct, mediators in AD pathogenesis [25].

Mechanistically, IL-4 and IL-13 signal through receptors that share the IL-4Rα subunit. IL-4 can engage either the type I receptor (IL-4Rα plus the common γ-chain) or the type II receptor (IL-4Rα plus IL-13Rα1), while IL-13 signals exclusively through the type II receptor [48]. Receptor binding activates Janus kinases: IL-4 activates JAK1 and JAK3 via the type I receptor, whereas both IL-4 and IL-13 activate JAK1 and TYK2 via the type II receptor [15,25]. This leads to phosphorylation of IL-4Rα and subsequent recruitment of STAT proteins, particularly STAT6. Activated STAT6 dimerizes and translocates to the nucleus, driving the transcription of genes associated with type 2 inflammation [49]. Additionally, IL-4 and IL-13 can activate STAT3 and alternative signaling pathways such as IRS-2/PI3K, which support keratinocyte survival and contribute to barrier dysfunction [48].

Although IL-4 and IL-13 share type 2 functions, they differ in their tissue distribution, functional hierarchy, and clinical relevance. IL-4 primarily acts in lymphoid organs, initiating type 2 immune responses by promoting Th2 differentiation and IgE class switching in B cells [50]. Through its upstream role in Th2 polarization, IL-4 contributes to the propagation of systemic type 2 inflammation beyond the skin and plays a central role in initiating and exacerbating the allergic march. This is particularly relevant in patients such as children or those with multiple allergic comorbidities including asthma and chronic rhinosinusitis with nasal polyps [15]. IL-13 functions as a peripheral effector cytokine in the skin, playing a central role in barrier disruption and pruritus—features it shares with IL-4—while uniquely driving tissue remodeling and dermal fibrosis, particularly in chronic AD [50,51]. In patients with persistent, lesion-predominant disease, selective IL-13 inhibition may provide a more targeted and skin-focused therapeutic option, especially when dual cytokine blockade is unnecessary or less well tolerated [15]. These complementary mechanistic profiles underscore the importance of tailoring cytokine blockade to individual disease phenotypes, rather than assuming uniform superiority of dual inhibition.

Dupilumab is a fully human IgG4 monoclonal antibody that targets IL-4Rα, thereby blocking IL-4 signaling via both type I (IL-4Rα/γc) and type II (IL-4Rα/IL-13Rα1) receptors, and IL-13 signaling via the type II receptor—resulting in dual inhibition of upstream and downstream type 2 pathways [13]. This broad blockade suppresses Th2 expansion, eosinophil recruitment, and IgE production, while restoring keratinocyte differentiation and barrier protein expression [52]. Clinically, this leads to improvements in skin barrier integrity, reductions in pruritus, and normalization of inflammatory biomarkers. Dupilumab also indirectly suppresses other type 2 mediators, including IL-5 and IL-31, and may offer additional benefits in patients with systemic atopy or multi-organ type 2 disease, such as asthma or allergic rhinitis [52].

Tralokinumab is a fully human IgG4κ monoclonal antibody that selectively neutralizes IL-13 by inhibiting its interaction with the type II receptor complex (IL-4Rα/IL-13Rα1) while sparing IL-4–mediated signaling through both type I and type II receptors [14]. This selective inhibition preserves upstream immune priming but blocks IL-13-driven peripheral effector functions, such as pruritus, tissue remodeling, and barrier impairment [15]. Clinically, tralokinumab has been shown to restore skin barrier gene expression, improve microbial diversity, and reduce Staphylococcus aureus colonization [15]. Its narrower mechanism of action may help minimize systemic immune modulation and may be especially appropriate for patients with skin-limited disease or those who experience IL-4-related adverse effects during dupilumab therapy.

The structural and functional differences between type I and type II IL-4 receptor complexes—along with their downstream signaling, effector functions, and therapeutic relevance in AD—are summarized in Table 1.

## 4. Clinical Efficacy and Safety of Dupilumab and Tralokinumab

### 4.1. Dupilumab

The clinical efficacy and safety of dupilumab across a wide spectrum of age groups have been demonstrated in multiple pivotal trials and their long-term extensions. Key trials in adults include the 16-week monotherapy studies LIBERTY AD SOLO 1 and 2, the 52-week combination therapy study LIBERTY AD CHRONOS (with topical corticosteroids), and LIBERTY AD CAFÉ, which focused on patients with prior inadequate response or intolerance to cyclosporin A. Long-term maintenance and safety were assessed in LIBERTY AD SOLO-CONTINUE and a 5-year open-label extension (OLE) study. The evidence is further expanded by comprehensive pediatric and adolescent trials, including LIBERTY AD ADOL (12 to <18 years), LIBERTY AD PEDS (6 to <12 years), and LIBERTY AD PRESCHOOL (6 months to <6 years).

In the SOLO 1 and 2 trials (N = 1379), dupilumab monotherapy resulted in significantly more patients achieving an Investigator’s Global Assessment (IGA) score of 0 or 1 compared to placebo at week 16 (36–38% vs. 8–10%, *p* < 0.001) [13]. In the 52-week CHRONOS trial (N = 740), which combined dupilumab with TCS, IGA 0/1 was achieved in 39% of patients in both dupilumab arms versus 12% in the placebo group, while Eczema Area and Severity Index 75% improvement (EASI-75) was achieved by 64–69% versus 23% for placebo (*p* < 0.0001) [53]. The exposure-adjusted incidence rate (EAIR) of serious treatment-emergent adverse events (TEAEs) was 4.05 (Q2W), 3.12 (QW), and 5.86 (placebo) per 100 patient-years [53]. In the CAFÉ study (N = 325) targeting CsA-experienced patients, EASI-75 responses were observed in 59.1% (QW) and 62.6% (Q2W), compared to 29.6% in the placebo group (*p* < 0.001) [54]. SOLO-CONTINUE (N = 422) demonstrated that EASI-75 was maintained at week 36 in 71.6% of patients continuing their original weekly or biweekly regimens, compared to 30.4% for those switched to placebo (*p* < 0.001) [55]. Long-term data from the 5-year adult OLE study (N = 2677) showed sustained efficacy, with 67.5% of patients maintaining an IGA 0/1 and 88.9% achieving EASI-75 at week 260 [56]. The EAIR of serious TEAEs was 6.66 events per 100 patient-years, with the incidence declining over time [56].

In adolescent patients (aged 12 to <18 years) in LIBERTY AD ADOL (N = 251), dupilumab achieved IGA 0/1 in 24.4% (Q2W) and 17.9% (Q4W) versus 2.4% in placebo, and EASI-75 in 41.5% (Q2W) and 38.1% (Q4W) versus 8.2% (placebo) (*p* < 0.001 for all comparisons) at week 16 [57]. In children aged 6 to <12 years (LIBERTY AD PEDS, N = 367), IGA 0/1 was achieved in 29.5–32.8% and EASI-75 in 67.2–69.7% with dupilumab regimens, compared to 11.4% and 26.8% in placebo, respectively (*p* ≤ 0.0004 for all) [58]. Among children aged 6 months to <6 years (LIBERTY AD PRESCHOOL Phase 3, N = 162), IGA 0/1 was achieved in 28% vs. 4% and EASI-75 in 53% vs. 11% (dupilumab vs. placebo, respectively; *p* < 0.0001) [59]. In the PED-OLE extension studies, efficacy and safety were sustained over 52 weeks. EAIRs of serious TEAEs were 1.6 (adolescents), 6.2 (6–11 years), and 6.8 (0.5–5 years) per 100 PYE [60,61,62]. EASI-75 responses were maintained in 81.2% (adolescents), 82% (6–11 years), and 79.3% (0.5–5 years) at 52 weeks [60,61,62].

In summary, these trials demonstrate the robust and durable efficacy of dupilumab across a wide range of age groups, from infants as young as 6 months to adults, with a consistent and acceptable long-term safety profile. A summary of the key study designs and primary outcomes is provided in Table 2.

### 4.2. Tralokinumab

The clinical efficacy and safety of tralokinumab have been established through the ECZTRA clinical trial program, including the ECZTRA 1 and 2 monotherapy studies, ECZTRA 3 and 7 combination therapy studies, and the ECZTRA 6 trial in adolescents. Long-term data are available from the ECZTEND OLE study. A summary of these trials is provided in Table 3.

In the ECZTRA 1 and 2 monotherapy trials (N = 802 and 794), tralokinumab 300 mg Q2W demonstrated significantly greater IGA 0/1 (15.8–22.2% vs. 7.1–10.9% placebo; *p* ≤ 0.002) and EASI-75 (25.0–33.2% vs. 12.7–11.4% placebo; *p* < 0.001) responses at week 16 [14]. Regarding safety, the incidence of serious adverse events at week 16 was comparable between the tralokinumab and placebo groups (1.7–3.8% vs. 2.5–4.1%, respectively) [14]. For patients who were responders at week 16 and continued Q2W dosing, EASI-75 was maintained at week 52 in 56–60% of patients [14]. In the TCS-adjuvant trial ECZTRA 3 (N = 380), IGA 0/1 and EASI-75 were achieved by 38.9% and 56.0% of patients receiving tralokinumab, respectively, compared to 26.2% and 35.7% in the placebo group at week 16 (*p* ≤ 0.015) [63]. In ECZTRA 7 (N = 277), which enrolled a CsA-refractory population, tralokinumab plus TCS demonstrated significantly higher EASI-75 rates compared to placebo plus TCS at week 16 (64.2% vs. 50.5%; *p* = 0.018) [64].

Adolescent patients (12–17 years) were evaluated in ECZTRA 6 (N = 289), where tralokinumab monotherapy led to significantly higher rates of IGA 0/1 (17.5–21.4% vs. 4.3% placebo; *p* ≤ 0.002) and EASI-75 (27.8–28.6% vs. 6.4% placebo; *p* < 0.001) at week 16 [65]. A pooled safety analysis of five RCTs (N = 2285) up to week 16 found that the EAIR of serious adverse events was lower for tralokinumab than placebo (7.4 vs. 11.9 per 100 PYE) [66]. Notably, rates of skin infections requiring systemic treatment were lower in the tralokinumab group (9.7 vs. 22.8 per 100 PYE), while conjunctivitis was more frequent (21.0 vs. 6.9 per 100 PYE) [66]. The ECZTEND OLE study demonstrated sustained long-term efficacy of tralokinumab, with 93% of patients achieving EASI-75 and 67% achieving IGA 0/1 over up to 5 years of treatment (N = 1672) [67], and an EAIR of serious adverse events of 6.7 per 100 PYE over up to 4.5 years of treatment (N = 2693) [68]. A head and neck subset analysis of ECZTEND showed the proportion of patients with head and neck EASI ≤ 1 increased from 12.2% at baseline to 87.2% at week 152 [69]. Post hoc analyses of ECZTRA 1 and 2 showed that among initial responders, a significant portion could maintain response on a Q4W dosing schedule, and 94.6% of those who relapsed on Q4W regained clinical response after reverting to Q2W dosing [70].

Taken together, these trials have established the efficacy and safety of tralokinumab in adults and adolescents with moderate-to-severe AD, with supportive long-term data demonstrating durable response and a consistent safety profile. A summary of the key study designs and primary outcomes is provided in Table 3.

## 5. Discussion and Practical Considerations

A direct comparison of dupilumab and tralokinumab reveals distinct clinical profiles that can guide personalized treatment selection. Pivotal monotherapy trial data indicates that dupilumab demonstrates numerically higher efficacy rates in achieving key endpoints such as IGA 0/1 and EASI-75 at 16 weeks (Table 4). However, long-term safety data from large OLE studies show that both agents possess a comparable and favorable safety profile, with similar rates of serious adverse events. The key differentiators emerge in their specific adverse event profiles and practical attributes. However, dupilumab is associated with a higher incidence of both conjunctivitis and injection-site reactions. These key distinctions, along with differences in head and neck efficacy, dosing flexibility, and administration methods, form the basis for a tailored therapeutic approach.

Recent clinical experience suggests that head and neck (H&N)-predominant AD may respond differently to biologic therapies. Several studies and real-world reports have described dupilumab-associated H&N dermatitis that persists or worsens despite overall clinical improvement, raising the possibility of regional disease heterogeneity and distinct immune endotypes [71,72,73]. Proposed mechanisms include Malassezia hypersensitivity, overlapping allergic contact dermatitis, and paradoxical Th1/Th17 activation in anatomically sensitive sites [71,72,73]. In this context, IL-13-selective inhibition with tralokinumab may represent a distinct therapeutic approach by preserving IL-4-mediated immune priming. Supporting this rationale, long-term extension data from the ECZTEND trial showed that the proportion of patients achieving H&N EASI ≤ 1 increased from 12.2% at baseline to 87.2% at week 152, with EASI subscore improvements comparable across body regions throughout the study [69]. Accordingly, tralokinumab may be preferred in patients with refractory H&N involvement or those experiencing worsening with dupilumab, although no head-to-head studies have yet established superiority in H&N-predominant AD.

Conjunctivitis has emerged as a key adverse event influencing the clinical choice between dupilumab and tralokinumab. At 16 weeks, conjunctivitis was reported in 9.3% of patients receiving dupilumab compared to 7.5% of those receiving tralokinumab, with dupilumab associated with a higher hazard ratio (HR 4.4 vs. 2.4) and a greater proportion of moderate-to-severe cases [74,75]. This pattern suggests a potential contribution of IL-4 blockade to conjunctivitis development, although further studies are warranted to delineate the specific roles of IL-4 and IL-13 in conjunctival homeostasis. Several hypotheses have been suggested, including reduced goblet cell density and mucus production after IL-4/IL-13 blockade or shifts in immune deviation (e.g., Th1/Th17-skewed responses), possibly in association with Demodex overgrowth or altered local immune regulation [74,75]. However, these mechanisms remain speculative, and further studies are warranted to clarify the pathogenesis of conjunctivitis in this setting.

In terms of injection-site reactions, while they are reported for both agents, published data suggest a numerically more favorable profile for tralokinumab. In 16-week pivotal monotherapy trials, injection-site reactions were reported in 15.4–15.8% of dupilumab-treated patients, compared to approximately 5.8% for tralokinumab [13,14]. This trend was also observed in long-term OLE data, where the exposure-adjusted event rate for injection-site reactions was lower for tralokinumab than for dupilumab (3.6 vs. 8.68 events per 100 PYE, respectively) [56,68].

Patient phenotype may also influence treatment choice. Elevated baseline IgE levels and comorbid type 2 conditions such as asthma or chronic rhinosinusitis with nasal polyps are indicative of systemic type 2 inflammation, for which dupilumab may be more effective due to its broader suppression of type 2 cytokine signaling through dual IL-4 and IL-13 blockade. In contrast, tralokinumab can be considered in patients with localized disease, intolerance to dupilumab (e.g., conjunctivitis), or when flexible dosing or cost is a concern. Notably, tralokinumab has shown sustained efficacy in patients with H&N involvement, suggesting its potential suitability for this phenotype. However, current evidence does not establish definitive superiority of one agent over the other, and further research is needed for optimal treatment selection in AD subgroups.

Recent North American real-world data from a registry of 857 patients showed that 56% achieved complete or near-complete itch resolution and 65% had no or minimal disease at 3 years with dupilumab, with no new safety concerns reported [76]. In a Central European registry (N = 220), 76% of dupilumab-treated patients achieved EASI-75 at week 16, maintained at 88% at weeks 28 and 40 [77]. In the interim TRACE real-world study (N = 650), tralokinumab treatment led to 57% achieving IGA 0/1 at 9 months, with head and neck involvement decreasing from 80% at baseline to 52% at 9 months, regardless of prior dupilumab use [78]. Recent European real-world data from a cohort of 30 patients showed a 60% EASI-75 response at week 16 with tralokinumab, with no serious adverse events and conjunctivitis reported in 10% (3/30) [79]. Similar data from another European cohort (N = 10) reported a 70% EASI-75 response at week 16, with no serious adverse events or conjunctivitis observed [80]. Collectively, these real-world findings highlight the effectiveness and safety of dupilumab and tralokinumab in routine clinical practice, underscoring the consistency of outcomes beyond controlled trial environments.

In real-world practice, selecting between dupilumab and tralokinumab for moderate-to-severe AD involves practical factors beyond efficacy and safety, such as administration convenience, dosing flexibility, cost, and patient-specific preferences. Dupilumab is available in both syringe and pen-type autoinjector formats, which may facilitate self-administration and improve adherence. Moreover, dupilumab has been in clinical use for a longer period, which may contribute to greater familiarity and prescribing confidence among both physicians and patients.

In contrast, tralokinumab is currently available only as a 150 mg prefilled syringe and requires two injections per standard 300 mg dose. However, it offers other practical advantages that may support adherence, including potentially lower cost in certain healthcare systems and flexible maintenance dosing intervals (every 2 or 4 weeks). Emerging real-world data further suggest that selected patients may achieve sufficient disease control with extended or reduced off-label regimens—such as single-syringe dosing or prolonged intervals—allowing for more personalized, cost-efficient treatment strategies.

## 6. Limitations

This review has several limitations. First, no head-to-head trials directly comparing dupilumab and tralokinumab have been conducted, limiting clinicians’ ability to make evidence-based treatment decisions between the two agents. Second, while both agents demonstrate overall favorable safety profiles, long-term safety data remain limited, particularly for tralokinumab. In particular, data on rare but serious adverse events—such as serious infections, immunologic complications, or potential malignancy risks—remain insufficient and require further surveillance. Third, most available data are derived from pivotal clinical trials, and real-world evidence comparing efficacy, safety, and adherence in broader patient populations remains lacking. Fourth, given the heterogeneity of atopic dermatitis, further studies are warranted to determine whether treatment efficacy differs across specific disease subtypes. Finally, although cost and access are important factors in real-world practice, comprehensive pharmacoeconomic analyses comparing these biologics in terms of cost-effectiveness are still lacking.

## 7. Conclusions and Future Directions

In conclusion, both dupilumab and tralokinumab have demonstrated substantial efficacy in the management of moderate-to-severe AD, with long-term safety data supporting their role as critical biologic therapies. Dupilumab, through its dual blockade of IL-4 and IL-13 signaling, has become a well-established treatment, while tralokinumab offers a more targeted approach by selectively neutralizing IL-13. The differences in their mechanisms of action, dosing regimens, and administration methods provide clinicians with important options for tailoring treatment to individual patient needs. A summary of key similarities and differences in mechanism, dosing, safety, and clinical applicability between dupilumab and tralokinumab is presented in Table 4. Importantly, there are currently no direct head-to-head trials comparing dupilumab and tralokinumab, and all efficacy and safety comparisons should be interpreted within this limitation.

Future research should aim to clarify the optimal positioning of advanced therapies, including biologics (e.g., dupilumab, tralokinumab, and lebrikizumab), JAK inhibitors (e.g., baricitinib, upadacitinib, and abrocitinib), and other emerging options. To address this, direct head-to-head trials are urgently needed to establish clear comparative efficacy and safety profiles. In parallel, the development and validation of biomarker-driven treatment algorithms are paramount to better delineate patient subgroups and inform personalized therapeutic strategies. Specifically, prospective validation is required to determine if baseline tissue/serum IL-13 levels can serve as a primary predictive marker for response to IL-13 inhibitors. Exploring other biomarkers linked to type 2 inflammation, such as IL-31, IL-33, serum thymus and activation-regulated chemokine (TARC), periostin, and dipeptidyl peptidase-4 (DPP-4), could also aid in predicting differential responses.

Beyond symptomatic control, whether these biologics can modify disease progression and alter the natural course of AD, including the atopic march, remains a critical area for future research. IL-4 inhibition, particularly with dupilumab, holds promise for modifying disease progression and potentially preventing allergic comorbidities. Long-term prospective studies, especially in pediatric populations, are essential to determine if sustained treatment can lead to lasting remission or prevent the development of associated allergic conditions. The impact of selective IL-13 blockade by tralokinumab on the atopic march also remains an important question for future studies.

Furthermore, personalized treatment strategies can be enhanced by investigating combination therapies—such as biologics with conventional or topical treatments—and by better understanding how to select therapies based on specific clinical factors. Factors such as comorbidities, the presence of head and neck involvement, and regional cost-effectiveness will be pivotal in tailoring the most appropriate therapy for each patient. Ultimately, this evidence-based, individualized approach will enable clinicians to optimize the management of AD, ensuring better patient outcomes.

## Figures and Tables

**Table 1:** Comparative roles of IL-4 and IL-13 in the pathogenesis of atopic dermatitis.

Category	IL-4	IL-13
The common role in AD pathogenesis	Perpetuate cutaneous inflammation, pruritus, and barrier dysfunction via shared STAT6-mediated signaling and by suppressing cutaneous antimicrobial peptides	
Expression in AD lesions	Low or inconsistent expression	Strong expression in acute and chronic AD lesions
Receptor signaling	Type I (IL-4Rα + γc) and Type II (IL-4Rα + IL-13Rα1)	Type II (IL-4Rα + IL-13Rα1)
Th2 differentiation	Strong promoter of naïve T cell to Th2 differentiation	Promotes Th2 differentiation (downstream of IL-4)
Tissue distribution	Primarily lymphoid organs (systemic)	Primarily skin (peripheral)
Fibrosis and remodeling	No major role	Promotes fibrotic skin remodeling
Atopic march relevance	Plays a key role, especially in children with asthma, CRSwNP	Minimal contribution; may exacerbate chronic itch/inflammation
IgE production	Activates B cells and promotes IgE class switching	Indirectly supports IgE via Th2 cytokines
Targeted by	Dupilumab (via IL-4Rα blockade)	Dupilumab (via IL-4Rα blockade), tralokinumab, lebrikizumab

AD (atopic dermatitis); IL (interleukin).

**Table 2:** Key clinical trials of dupilumab in moderate-to-severe atopic dermatitis.

Ref.	Study Title	Age Group	Study Design/Duration	Patients (N)	Key Efficacy Outcome	Key Safety Outcome (SAEs)
[13]	LIBERTY AD SOLO 1 & 2	Adults	16-week RCT (monotherapy)	1379	IGA 0/1: 36–38% vs. 8–10% (*p*< 0.001) EASI-75: 44–52% vs. 12–15% (*p* < 0.001)	1.8–1.9% vs. 3.4–4.5%
[53]	LIBERTY AD CHRONOS	Adults	52-week RCT with TCS	740	IGA 0/1: 39% vs. 12% (*p* < 0.0001) EASI-75: 64–69% vs. 23% (*p* < 0.0001)	EAIR: 4.05 vs. 5.86 / 100 PYE
[54]	LIBERTY AD CAFÉ	Adults	16-week RCT with TCS (CsA-failure)	325	IGA 0/1: 39–40% vs. 14% (*p* < 0.001) EASI-75: 59–63% vs. 30% (*p* < 0.001)	1.9% vs. 1.9%
[56]	LIBERTY AD OLE	Adults	Up to 5-year OLE	2677	IGA 0/1: 67.5% EASI-75: 88.9%	EAIR: 6.66/100 PYE
[57]	LIBERTY AD ADOL	12–17 years	16-week RCT (monotherapy)	251	IGA 0/1: 24.4% vs. 2.4% (*p* < 0.001) EASI-75: 41.5% vs. 8.2% (*p* < 0.001)	0% vs. 1.2%
[58]	LIBERTY AD PEDS	6–11 years	16-week RCT with TCS	367	IGA 0/1: 30–33% vs. 11% (*p* < 0.001) EASI-75: 67–70% vs. 27% (*p* < 0.0001)	0% vs. 1.7%
[59]	LIBERTY AD PRESCHOOL	0.5–5 years	16-week RCT with TCS	162	IGA 0/1: 28% vs. 4% (*p* < 0.0001) EASI-75: 53% vs. 11% (*p* < 0.0001)	No new safety signals identified

Abbreviations: CsA, cyclosporin A; EAIR, exposure-adjusted incidence rate; EASI-75, ≥75% improvement in Eczema Area and Severity Index; IGA, Investigator’s Global Assessment; OLE, open-label extension; PYE, patient-years of exposure; RCT, randomized controlled trial; SAE, serious adverse event; TCS, topical corticosteroids.

**Table 3:** Key clinical trials of tralokinumab in moderate-to-severe atopic dermatitis.

Ref.	Study Title	Age Group	Study Design/Duration	Patients (N)	Key Efficacy Outcome	Key Safety Outcome (SAEs)
[14]	ECZTRA 1 & 2	Adults	16-week RCT (monotherapy)	1596	IGA 0/1: 15.8–22.2% vs. 7.1–10.9% (*p* ≤ 0.002) EASI-75: 25.0–33.2% vs. 11.4–12.7% (*p* < 0.001)	1.7–3.8% vs. 2.5–4.1%
[63]	ECZTRA 3	Adults	32-week RCT with TCS	380	IGA 0/1: 38.9% vs. 26.2% (*p* = 0.015) EASI-75: 56.0% vs. 35.7% (*p* < 0.001)	0.8% vs. 3.2%
[64]	ECZTRA 7	Adults	26-week RCT with TCS (CsA-failure)	277	EASI-75: 64.2% vs. 50.5% (*p* = 0.018)	0.7% vs. 3.6%
[65]	ECZTRA 6	12–17 years	52-week RCT (monotherapy)	289	IGA 0/1: 17.5–21.4% vs. 4.3% (*p* ≤ 0.002) EASI-75: 27.8–28.6% vs. 6.4% (*p* < 0.001)	1.0–3.1% vs. 5.3%
[66]	Pooled Safety (5 RCTs)	Adults	16-week Pooled Data	2285	N/A	EAIR: 7.4 vs. 11.9/100 PYE
[67]	ECZTEND OLE	≥12 years	Up to 5-year OLE	1672	IGA 0/1: 66.7% EASI-75: 92.9%	N/A
[68]	ECZTEND OLE	≥12 years	Up to 4.5-year OLE	2693	N/A	EAIR: 6.7/100 PYE
[69]	ECZTEND (H&N subset)	Adults	4-year OLE post hoc analysis	1192	H&N EASI ≤ 1 increased from 12.2% at baseline to 87.2% at Wk 152.	Paradoxical H&N erythema was rare (1.0%) and mostly resolved.
[70]	Q4W Analysis (ECZTRA 1/2)	Adults	52-week post hoc analysis	337	94.6% of patients who relapsed on Q4W regained response after reverting to Q2W.	Immunogenicity potential was low and not increased with Q4W dosing.

Abbreviations: CsA, cyclosporin A; EAIR, exposure-adjusted incidence rate; EASI, Eczema Area and Severity Index; H&N, head and neck; IGA, Investigator’s Global Assessment; N/A, not available; OLE, open-label extension; PYE, patient-years of exposure; Q2W, every 2 weeks; RCT, randomized controlled trial; SAE, serious adverse event; TCS, topical corticosteroids; Wk, week.

**Table 4:** Comparison of dupilumab and tralokinumab in moderate-to-severe atopic dermatitis.

Category	Dupilumab	Tralokinumab
Mechanism	Blocks signaling of both IL-4 & IL-13	Selectively neutralizes only IL-13
Indications in AD	Adults, adolescents, children and infants (≥6 months)	Adults, adolescents (≥12 years)
Indications beyond AD	Various *	None currently approved
Dosing	Initial: 600 mg (2 × 300 mg), then 300 mg Q2W	Initial: 600 mg (4 × 150 mg), then 300 mg Q2W; Q4W possible after 16 weeks
Dosing flexibility	Q2W maintenance fixed	Q2W/Q4W maintenance options
Efficacy from pivotal study (16 week) (monotherapy, adults)	Relatively higher (EASI-75: 44–52%)	Relatively lower (EASI-75: 25–33%)
Long-term safety from OLE studies	Data robust up to 6 years Favorable, rare SAEs (declining with time) SAE: 6.66/100 PY up to 5 years	Data robust up to 6 years Favorable, rare SAEs (declining with time), SAE: 6.7/100 PY up to 4.5 years
Common AE (Conjunctivitis)	15.34 per 100 PY	8.3 per 100 PY
Common AE ‡ (Injection site reaction)	15.4–15.8% (at 16 weeks) 8.68 per 100 PYE (OLE)	~5.8% (at 16 weeks) 3.6 per 100 PYE (OLE)
Device convenience	Pen and syringe available; requires single injection per dose	Syringe only; requires 2 injections per dose
Cost (region-dependent)	Often higher (variable by health system)	Often lower (variable by health system)

Abbreviations: AD, atopic dermatitis; AE, adverse event; EASI-75, ≥75% improvement in Eczema Area and Severity Index; IL, interleukin; OLE, open-label extension; PYE, patient-years of exposure; Q2W, every 2 weeks; Q4W, every 4 weeks; SAE, serious adverse event. * Asthma (type 2 inflammation-associated, moderate-to-severe), chronic rhinosinusitis with nasal polyps (CRSwNP), eosinophilic esophagitis (EoE), prurigo nodularis, chronic obstructive pulmonary disease (COPD, type 2 inflammatory subtype). SAE rate derived from pooled analysis of five randomized-controlled trials, however exact duration of exposure not specified. ‡ Tralokinumab OLE data reports ‘Injection site reaction’ (IR: 3.6) and ‘Injection site pain’ (IR: 1.5) as separate items. This table uses the value for the representative term ‘Injection site reaction’ for a like-for-like comparison.
